# Profiled Ion-Exchange Membranes for Reverse and Conventional Electrodialysis

**DOI:** 10.3390/membranes12100985

**Published:** 2022-10-11

**Authors:** Sergey Loza, Natalia Loza, Natalia Kutenko, Nikita Smyshlyaev

**Affiliations:** Physical Chemistry Department, Faculty of Chemistry and High Technologies, Kuban State University, 350040 Krasnodar, Russia

**Keywords:** profiled ion-exchange membrane, reverse electrodialysis, current-voltage curve, conductivity, diffusion permeability, microheterogeneous model, selectivity

## Abstract

Profiled ion-exchange membranes are promising for improving the parameters of reverse electrodialysis due to the reduction of pumping power and electrical resistance. The smooth commercial heterogeneous cation-exchange MK-40 and anion-exchange MA-41 membranes were chosen as the initial membranes. Profiled membranes with three different types of surface profiles were obtained by hot pressing the initial membranes. The bilayer membranes were made on the basis of single-layer profiled membranes by casting MF-4SK film on the profiled surfaces. The diffusion permeability of all types of single-layer and bilayer profiled membranes was higher than of the initial ones due to the appearance of large defects on their surface during pressing. The conductivity of the profiled membrane was lower in the diluted solution and higher in the concentrated solution than of the initial one for all samples except for the bilayer anion-exchange membrane. The conductivity of that sample was lower than that of the initial anion-exchange MA-41 membrane over the entire range of studied concentrations. The counter-ion transport numbers for all studied membranes were calculated based on the concentration dependences of conductivity and diffusion permeability of the membrane by the microheterogeneous model. The selectivity of single layer and bilayer profiled membranes became lower after their profiling due to the increase of the solution phases of membranes. The asymmetry of the current-voltage curves for all single-layer and bilayer profiled membranes was found. The application of the single layer and bilayer profiled membranes in reverse electrodialysis did not lead to an increase in power density.

## 1. Introduction

At the present time, a big demand for the development of alternative ways of energy production comes from the global economy. It is constantly growing due to the need to reduce the use of fossil fuels. One of the promising sources of renewable energy is the energy obtained by mixing two salt fluxes of different concentrations [1,2,3,4,5,6,7]. Theoretically, electrical power of 2400 GW can be obtained by mixing the runoff of all rivers with ocean water, but only 40% of this power can be technically utilized [8]. This potential can be used in coastal areas by mixing river water with sea water using reverse electrodialysis (RED).

Although the principle of RED technology has been known for almost 70 years [9], significant efforts are required to study the real potential of such a process. The RED stack consists of alternately stacked cation-exchange membranes (CEMs) and anion-exchange membranes (AEMs) and is used to get the Gibbs energy released by mixing solutions of various concentrations. The power characteristic of the RED process depends on the membranes properties [1,3,10,11,12], the electrolyte solutions flow velocities across the RED stack [2,13,14,15,16], salinity ratio [13,14,16,17], the construction peculiarities of the RED stack [13,18], the solutions composition [16,19,20], and the temperature or its gradient [20,21].

The main problems of RED are the relatively low net power density per membrane area and the high cost of electricity generated due to the cost of the ion-exchange membranes (IEMs). Therefore, it is necessary to use highly efficient and inexpensive membrane materials for RED [7,22]. The lower the electrical resistance of the RED stack and pumping power are, the higher the net power density is. The nonconducting spacers are used to separate the neighboring membranes, but it leads to an increase in the electrical resistance and a decrease in the IEMs area available to counter-ion flux due to the shadow effect. Besides, spacers are the main source of hydraulic resistance compared to empty channels [6,8]. The use of the profiled membranes instead of smooth ones allows the refusal to abandon the use of nonconducting spacers. Also, there is an increase in the received power as a result of a decrease in the hydrodynamic resistance [6,23,24,25,26]. In addition, profiling heterogeneous IEMs can significantly improve their electrochemical characteristics, such as conductivity [27]. However, Gurreri et al. have theoretically shown [28] that the use of profiled membranes does not always lead to an improvement in RED performance, depending on stack features and operating conditions.

It is known that the structure of the membrane surface has an important influence on the transport of ions and molecules in the processes of conventional electrodialysis (ED), RED, and dialysis [29,30,31,32,33,34]. V.V. Nikonenko and co-authors have shown [35,36] that the microheterogeneity of the membrane surface contributes to the intensification of mass transfer in overlimiting current modes due to the electroconvection development. In addition to geometric inhomogeneity, which has a large effect on ion transfer during ED [37], the ratio of conductive and non-conductive areas on the membrane surface also influences the electroconvection development [38]. This ratio depends on the content of ion-exchange resin [39] and its dispersion [40]. Geometric inhomogeneity of the membrane surface affects its basic properties, such as conductivity and diffusion permeability [27,41]. On the one hand, the study of the influence of the membrane surface profile on the efficiency of dialysis separation has shown that the replacement of a smooth membrane with a profiled one leads to an increase in the amino acid flux due to its facilitated diffusion and a decrease in a mineral salt flux because of Donnan exclusion [42]. On the other hand, an increase in the diffusion permeability of profiled membranes in comparison with smooth ones negatively affects the RED and ED. This problem can be solved by applying a conductive film of a Nafion-type homogeneous polymer on the membrane profiled surface [36]. Besides, it might reduce membrane fouling during RED [22,29] and provide the single charged ion permselectivity for the AEMs [29,43,44]. However, the prospects of the application of profiled membranes with the layer of a conductive polymer film (profiled bilayer membrane) in the RED are still unclear.

The selectivity of membranes has a significant effect on the magnitude of the electromotive force arising in the RED stack according to the formula [45]

(1) $$\Delta E_{OCV}=2N_{mp}\frac{t_{av}RT}{F}ln\frac{a_{c}}{a_{d}},$$

where *ΔE_OCV_* is the open-circuit potential; *R* is the universal gas constant; *T* is the absolute temperature; *F* is the Faraday constant; *a_d_* and *a_c_* are the activities of diluted and concentrated electrolyte solutions, respectively; *N*_mp_ is the number of membrane pairs, and *t_av_* is the average selectivity of the membrane pair. The membrane selectivity is described by the counter-ion transport number [32,46,47].

There are several ways to obtain the counter-ion transport number in a membrane, each of which has its peculiarities [48,49,50]. The transport number obtained by the potentiometric method does not account for the water transport through the membrane and is called the apparent ion transport number. In this case, it is necessary to measure the electroosmotic permeability of a membrane and calculate the “true” ion transport number by the well-known Scatchard’s equation [48]. The water transport number is traditionally used to describe the electroosmotic permeability of a membrane. There are two methods for obtaining the water transport number for IEM. Both of them are very difficult, laborious, and time-consuming [51,52,53]. Using the gravimetric method, the electrode reactions should be taken into consideration. Besides, the experimental error in determining the solution weight might be significant. Another method allows evaluating the water transport values only for solutions that contain the chloride anions because only reversible Ag/AgCl electrodes can be used for this experiment. Another way to determine the counter-ion transport number is by measuring the flux of all the ions transported through the CEMs and AEMs under the direct current [50,54,55,56]. However, it is also difficult and time-consuming to do routine experiments and needs a lot of equipment to provide correct experimental results. Besides, like the potentiometric method, this one must consider the water transport through the membrane. The chronopotentiograms of IEM make it possible to determine the ion transport number from the transition time [57,58,59].

A two-phase microheterogeneous model can be used to estimate the counter-ion transport number of a membrane by the formula [60,61,62]

(2) $$t_{counter}^{*}=\frac{z_{counter}^{2}L_{counter}^{*}}{z_{counter}^{2}L_{counter}^{*}+z_{co}^{2}L_{co}^{*}},$$

where $t_{counter}^{*}$ is the counter-ion transport number, *z_counter_* and *z_co_* are the number of counter- and co-ion charges, respectively, and $L_{counter}^{*}$ and $L_{co}^{*}$ are the phenomenological electrodiffusion coefficients of counter- and co-ions, respectively. The phenomenological electrodiffusion coefficients and counter-ion transport numbers are calculated based on the concentration dependences of the membrane conductivity and diffusion permeability. A systematic study of the properties of basic industrial membranes and the identification of the effects of temperature and pressure on them, as well as the subsequent application of a layer of sulfonated polymer, allows us to create approaches to optimizing the properties of commercial materials by modifying them.

Thus, the aim of this work is to assess the type of surface profile of CEMs’ and AEMs’ effect on their basic properties, such as conductivity, diffusion permeability, and selectivity, as well as the efficiency of the application of profiled single layer and bilayer membranes in the process of electrical energy generation by the RED.

## 2. Materials and Methods

### 2.1. Membranes

The MK-40 CEM and MA-41 AEM (Ltd. “Innovative Enterprise Shchekinoazot,” Tula Region, Russia) were used as the initial membranes. Both membranes are heterogeneous materials, consisting of ion-exchange resins, polyethylene, and reinforcing polyamide mesh. Ion-exchange resins are polystyrene crosslinked by divinylbenzene containing the sulfo groups as fixed ions in the case of MK-40 membrane and the mainly quaternary amines and small amounts of secondary and tertiary amines as fixed groups in the case of MA-41 membrane. Table 1 shows the main characteristics of initial and modified membranes. The ion-exchange capacity (IEC) was measured for H^+^-form samples in the case of the MK-40 membranes and for OH^−^-form in the case of MA-41. The MK-40 and MA-41 membranes were immersed in solutions containing the excess of NaOH or HCl, respectively. OH^−^ or H^+^ ion concentration in studied solutions was determined by acid-base titration after 24 h, and the value of IEC was calculated as follows

(3) $$IEC=\frac{V\Delta C}{m_{dry}},$$

where *V* was the studied solution volume, Δ*C* was the difference between the initial and final OH^−^-ions and H^+^-ions concentration in the studied solution for the MK-40 and MA-41 membranes, respectively, and *m_dry_* was the mass of the dry membrane [63]. The water uptake was measured by the gravimetric method [64]. The membrane thickness (*l*) and the water uptake were obtained for the membranes in Na^+^- and Cl^−^-ion forms. The specific water content, *n_w_*, was calculated as the ratio of the water uptake to the *IEC* value. 

**Figure 1:**
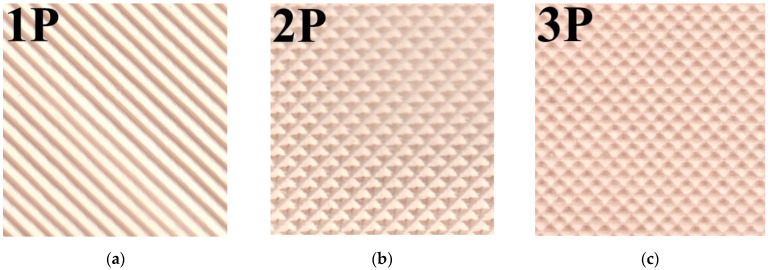
Images of profiled surfaces of IOMs, where (**a**) is profile No. 1, (**b**) is profile No. 2, and (**c**) is profile No. 3.

The profiled membranes were prepared by the hot pressing method described in [37]. The main peculiarity of this method consists of using a wet membrane. Profiled membranes were obtained under a pressure of 13 MPa, a temperature of 90 °C, and a holding time of 30 s. Three types of press forms were applied to obtain the 3 types of profiled membranes (Figure 1). The first type of profile (Figure 1a) had alternating stripes of a semicircular cross-section with a height of 0.35 mm and a distance of 1.5 mm between them. The second type of profile (Figure 1b) had a depressed tetrahedral pyramid with a base size of 1.5 × 1.5 mm (visually similar to a waffle print). The third type of profile (Figure 1c) had convex hemispheres with a 1 mm base diameter at a distance of 1.5 mm from each other. Profiled samples were marked by the number of a press form and the letter “P,” e.g., MK-40_1P or MA-41_1P.

The profiled membranes were used to prepare the bilayer membranes with the sulfonated perfluoropolymer MF-4SK layer on the profiled surface. A mixture of a 10% solution of sulfonated LF-4SK perfluoropolymer in isopropanol (JSC Plastpolymer, St. Petersburg, Russia) and acetic acid was cast on the profiled surface of the membranes according to the method described in detail in [37,65]. These samples were marked by the number of a profile type and letters “PM” (profiled and modified), e.g., MK-40_1PM or MA-41_1PM. Thus, two series of modified membranes were obtained: single-layer CEMs and AEMs with 3 types of profiled surfaces and bilayer ones with a homogeneous MF-4SK film. 

After preparation, all the membranes were pretreated by the standard procedure [48,64,66], including surface cleaning with CCl_4_ for degreasing, immersion in ethanol for 6 h to remove monomer and oligomer residues from the ion-exchange resin, and sequential immersion in 300 g/L, 100 g/L, 30 g/L NaCl solutions for 24 h in each step for gradual swelling, swollen membranes were washed by deionized water.

### 2.2. Scanning Electron Microscopy

The studies of the surface morphology of the dry membranes were carried out by low vacuum scanning electron microscopy (SEM) using a JSM-7500 scanning electron microscope (JEOL, Tokyo, Japan). A thin carbon layer was deposited on the surface of the membranes before the study.

### 2.3. Conductivity and Diffusion Permeability Measurements

The conductivity of IEMs (*k_m_*) was obtained from the active part of the membrane AC resistance measured by the mercury contact method [67] by the formula

(4) $$k_{m}=\frac{l}{R_{m}\cdot S},$$

where *R_m_* was the membrane resistance and *S* was the membrane area. The resistance was measured using the potentiostat-galvanostat Autolab PGSTAT302N equipped with an FRA-32 impedance unit (Metrohm Autolab B.V., Utrecht, The Netherlands). 

**Figure 2:**
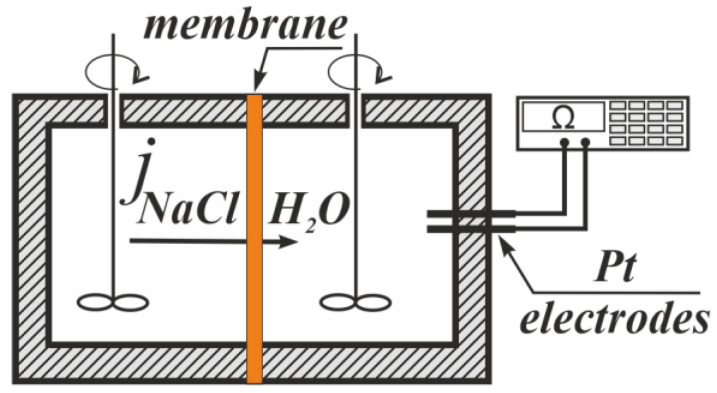
The experimental setup for the diffusion permeability measurement.

The diffusion permeability of IEMs was obtained using a non-flow two chambers cell (Figure 2) using the method described in [66]. The studied membrane separated two chambers. The first chamber contained a studied solution, whereas deionized water and platinum electrodes for the resistance measurement were in the second. The solutions in both chambers were stirred. The solution resistance value in the second chamber depended on the quantity of electrolytes transferred through the membrane.

The time dependence of conductance in the second chamber was used to calculate the flux density (*j_m_*) and integral diffusion permeability coefficient (*P_m_*) by the formulae

(5) $$j_{m}=\frac{V}{S}K\frac{d(\frac{1}{R_{s}})}{d\tau },$$

(6) $$P_{m}=j_{m}\frac{l}{C_{0}},$$

where *V* was the chamber volume (100 mL), *R_s_* was the resistance in the second chamber, *τ* was the time of the experiment, *C_0_* was the electrolyte concentration in the first chamber, and *K* was the constant of the diffusion cell that depends on the geometry of the electrodes and the electrolyte nature. The *K* value was obtained as a slope of dependence in coordinates C-(1Rs).

### 2.4. Current-Voltage Curve Measurements

The current-voltage curve (CVC) of the membrane was measured in 0.05 mol/L NaCl solutions by the method described elsewhere [68]. The galvanodynamic regime was used for the CVC measurements at a scanning rate of 10^−4^ A/s. Direct current was applied to platinum polarizing electrodes using the Keithley 2420 SourceMeter (Keithley Instruments, Inc., Cleveland, OH, USA). The potential drop across the membrane under study was measured using Ag/AgCl electrodes by the Keithley 2701 Ethernet Multimeter/Data Acquisition System (Keithley Instruments, Inc., Cleveland, OH, USA). These electrodes were placed in the Luggin–Haber capillaries, which were installed on both sides of the studied membrane in its geometric center at a distance of about 0.5 mm from the surface. The solution circulated through each chamber from individual storage tanks at a flow rate of 14 mL/min.

**Figure 3:**
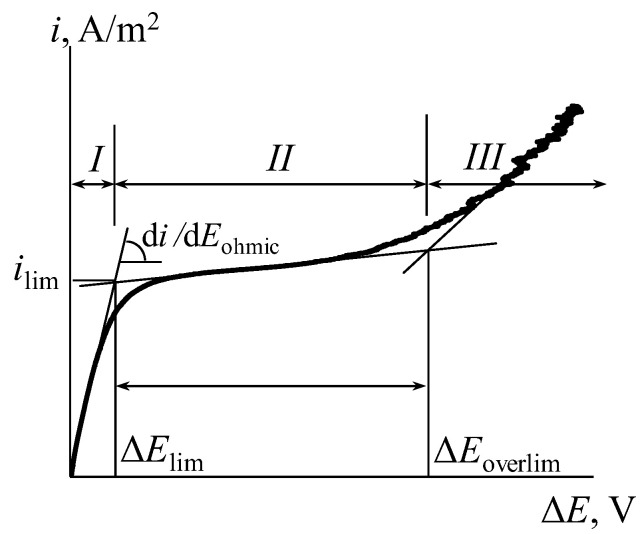
Typical CVC of the IEM: *I:* the ohmic section; *II:* the plateau of limiting current; *III:* the overlimiting region.

The parameters of CVC were calculated graphically: the slope of the ohmic section of the CVC (*d_i_/dE_ohmic_*, S/m^2^), the magnitude of the limiting diffusion current density (*i_lim_*, A/m^2^), the voltage drops of the transition of the IEM into the limiting (*ΔE_lim_*, V) and the overlimiting (*ΔE_overlim_*, V) state (Figure 3, sections *I* and *III* respectively) and the length of the limiting current density plateau (*Δ*, V; Figure 3, section *II*). In the case of a noticeable slope of the limiting current density plateau, the limiting diffusion current density was calculated using numerical differentiation. 

The IEMs were equilibrated with the studied solutions before all measurements (conductivity, diffusion permeability, and CVC measurements). All experiments were carried out at the constant temperature of 25 °C and managed at least 3 times. The average values, standard deviations, confidence intervals, and absolute errors were calculated for all experimental data using standard features of Microsoft Excel. The *k_m_*, *P_m,_* and *j_m_* determination errors were under 5% for the smooth membranes and under 10% for the profiled and bilayer ones. The *i_lim_* determination error was under 5%. Other parameters of CVC determination errors were under 10%. The diffusion permeability and CVC measurements were performed for both membrane orientations when the profiled or initial membrane surface is placed toward salt or counter-ion flow. The mark “*s*” is used to indicate the orientation of the membrane by the profiled surface towards the flow of salt or counter-ions. The mark “*w*” is used for the opposite membrane orientation.

### 2.5. RED Measurements

**Figure 4:**
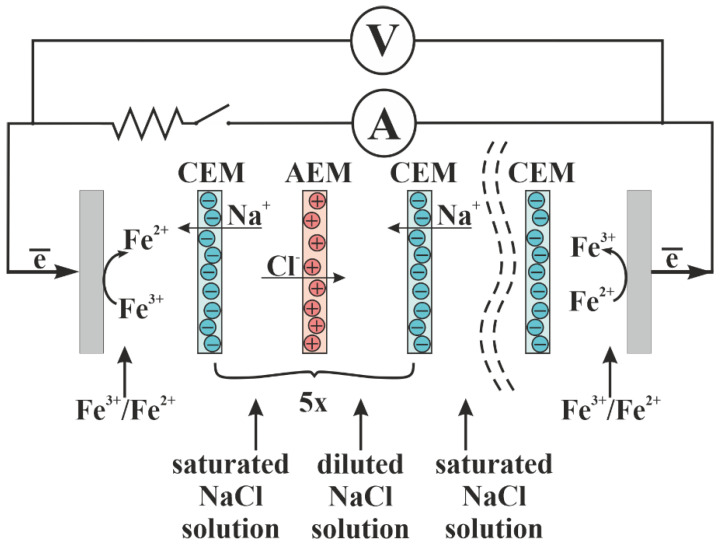
The experimental setup for the RED measurement. “5x” means that this element was repeated 5 times.

The experimental setup included the laboratory RED stack, peristaltic pumps, a set of resistors with a range of 0.5–500 Ohm, and the Agilent U1251B multimeters (Agilent Technologies, Santa Clara, CA, USA) for measuring voltage and current (Figure 4). The laboratory RED stack consisted of 10 chambers, each of which was formed by CEMs and AEMs, and 2 electrode chambers. Platinum-coated titanium electrodes were used. Membranes were separated by polyethylene gaskets, the thickness of which was 0.9 mm. Inert nylon mesh spacers were placed between the smooth membranes. A mixed solution of 0.025 mol/L K_4_[Fe(CN)_6_] and 0.025 mol/L K_3_[Fe(CN)_6_] in 0.25 mol/L NaCl circulated through the cathode and anode chambers sequentially. CEMs separated the electrode chambers from other chambers to prevent the migration of ferri-/ferro-cyanide anions. Concentrated and diluted solutions of sodium chloride were pumped alternately through adjacent chambers. 

A saturated sodium chloride solution circulated through the first chamber. The solution was changed after each measurement. Diluted sodium chloride was pumped through the adjacent chamber. The concentration of the solution was kept constant in each experiment. The concentration of the diluted sodium chloride solution varied from 2 mg/L (deionized water) to 19 g/L, which roughly corresponds to the salinity of the Black Sea water. Thus, there were five chambers with the saturated sodium chloride solution and 5 chambers with the diluted sodium chloride solution. The working size of each membrane was 5 × 20 cm^2^. The distance between the membranes was 1 mm. The volumetric circulation rate of the solutions was 8.57 L/h, which corresponded to a linear velocity of 0.013 m/s through each chamber of the RED stack. 

The voltage values with an open external circuit were recorded every 2 min until a constant voltage value was reached. This voltage was called the open circuit voltage (OCV). Then the external circuit, including the ammeter and a resistor with a maximum resistance of 500 Ohm, was closed, and the values of the current and voltage were measured. Then, every 2 min, the resistor was replaced by another one with a lower resistance, and the values of the current and voltage were also recorded. The last values of current and voltage were measured in the mode of short circuit of the RED stack. The specific power (Powers) for each resistance was calculated using the formula

(7) $$Power_{s}=\frac{IU}{SN},$$

where *I* was the current, *U* was the voltage, and *N* was the number of membranes in the RED stack.

**Figure 5:**
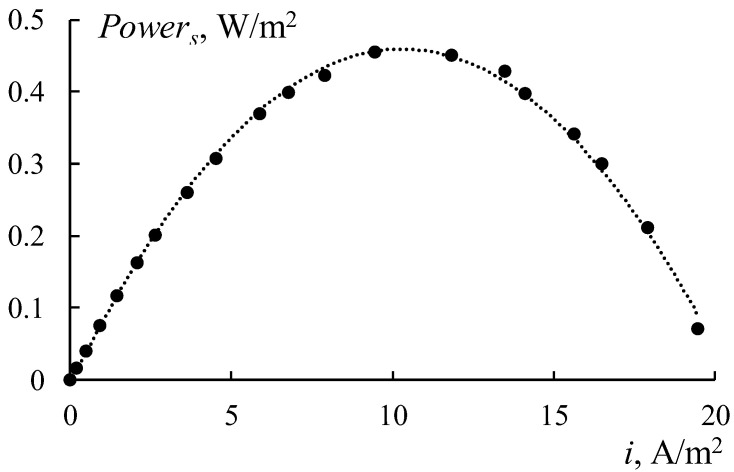
The dependence of the power density of the RED stack on the current density.

A typical dependence of the specific power of the RED stack on the current density is shown in Figure 5. The maximum value of the specific power was determined for each concentration ratio and was used to obtain the dependence of the maximum specific power of the RED stack on the concentration of a diluted sodium chloride solution. 

Based on this dependence, the optimal ratio of the solution concentrations for the estimation of the maximum value of the specific power was determined.

## 3. Theoretical Background

According to the microheterogeneous model, there are two conducting phases in the membrane structure [60,61,62]. The first phase, which is named the gel phase, consists of the membrane polymer matrix. In the diluted solution, if the membrane is highly selective (“ideally” selective), the conductivity of the gel phase is provided only by counter-ions, which quantity is equal to the number of fixed ions. The second phase includes the internal solution, which is equal to the external solution in compositions and properties, and it is called the intergel or solution phase. The general electrodiffusion properties of the IEM are described by the formula

(8) $$L_{m}={\left[f_{1}L_{1}^{\alpha }+f_{2}L_{2}^{\alpha }\right]}^{1/\alpha },$$

where *L_m_* is the electrodiffusion coefficient which is equal to conductivity or differential coefficient of diffusion permeability of the entire membrane; *f*_1_ and *f*_2_ are volume fractions of the gel and intergel phases, respectively, and *f*_1_ + *f*_2_ = 1; *L_1_* and *L_2_* are the phenomenological electrodiffusion coefficients which are equal to the conductivity or differential coefficient of diffusion permeability of the gel and intergel phases, correspondingly; *α* is a structural parameter characterizing the spatial orientation of the phases inside the membrane (it might possess the value in the range from −1 to 1 corresponding to serial and parallel orientation of conducting phases).

On the whole, the migration flux is limited by the counter-ion flux, and the diffusion flux is limited by the co-ion flux. The conductivity and differential coefficient of diffusion permeability of the membrane (P^*^) can be described by the formulae

(9) $$k_{m}={\left[f_{1}k_{iso}^{\alpha }+f_{2}k^{\alpha }\right]}^{1/\alpha },$$

(10) $$P*={\left[f_{1}{\left(GC\right)}^{\alpha }+f_{2}D^{\alpha }\right]}^{1/\alpha },$$

where *k_iso_* and *k* are the conductivities of the gel and intergel phases correspondingly, *G* is the complex parameter that describes the diffusion properties of the gel phase relating to co-ions, and *D* is the electrolyte diffusion coefficient in solution. 

The *G* parameter includes the membrane properties that are the most difficult to determine and can be described as follows

(11) $$G=K_{D}{\bar{D}}_{co}/\bar{Q},$$

(12) $$G=\frac{P^{*}}{C}{\left(\frac{\beta -1}{f_{1}}\right)}^{1/\alpha },$$

where *K_D_* is the Donnan constant, ${\bar{D}}_{co}$ is the diffusion coefficient of the co-ions in the gel phase of the membrane, $\bar{Q}$ is the ion-exchange capacity of the gel phase, and $\bar{Q}=Q/f_{1}$. Parameter *G* can be calculated by the Formula (12) if the concentration dependence of the diffusion permeability is increasing and the *β* value is higher than 1.

Otherwise, the *G* parameter is obtained by the Formula (11). However, the experimental determination of the Donnan constant and the diffusion coefficient of the co-ions in the gel phase of the membrane is very difficult, which limits the use of Formula (11) for the G parameter calculation. 

Parameter *α* tends to zero for most membranes [48,60], which corresponds to the chaotic distribution of conducting phases, and the Formula (9) is converted to

(13) $$k_{m}=k_{iso}^{f_{1}}\times k^{f_{2}},$$

The transport-structural parameters (*f*_1_ and *f*_2_, *α*, *G*) are estimated using the concentration dependences of the conductivity and diffusion permeability of a membrane. The *k_iso_*, *f_1,_* and *f*_2_ parameters are determined from the dependence *log* *k_m_* = *f* (*log* *k*) by the formula

(14) $$logk_{m}=f_{1}\times logk_{iso}+f_{2}\times logk,$$

where *f*_2_ is the slope of this dependence and *k_iso_* is calculated from $f_{1}\times logk_{iso}$.

The counter-ion transport number is calculated by the Formula (2). It includes the $L_{counter}^{*}$ and $L_{co}^{*}$ phenomenological electrodiffusion coefficients, which are calculated by the formula

(15) $$L_{counter}^{*}=\frac{k_{m}^{dc}}{2F^{2}}\left[1+\sqrt{1-\frac{2P^{*}CF^{2}}{RTk_{m}^{dc}\pi_{\pm }}}\right],$$

(16) $$L_{co}^{*}=\frac{k_{m}^{dc}}{2F^{2}}\left[1-\sqrt{1-\frac{2P^{*}CF^{2}}{RTk_{m}^{dc}\pi_{\pm }}}\right],$$

where $k_{m}^{dc}$ is the membrane conductivity under direct current, *π_±_* is the correction factor for the solution nonideality, and P^*^ is the differential coefficient of the diffusion permeability of the membrane. The correction factor for the solution nonideality is calculated by

(17) $$\pi_{\pm }=1+\frac{dln\gamma_{\pm }}{dlnC},$$

where *γ_±_* is the average ionic activity coefficient of the electrolyte solution.

The $k_{m}^{dc}$ is calculated by

(18) $$k_{m}^{dc}=k_{m}t_{counter}^{f_{2}},$$

where *t_counter_* is the counter-ion transport number in the electrolyte solution. The differential coefficient of the diffusion permeability of the membrane is calculated by the formula

(19) $$P^{*}=P_{m}\times \beta ,$$

where *β* is the model parameter, which is defined as the slope of the dependence *log* *j_m_* = *f* (*log* *C*). 

Obviously, the density of the diffusion flux through the membrane always grows with an increase in the concentration of the electrolyte solution. However, the differential coefficient of the membrane diffusion permeability can both increase and decrease with increasing solution concentration. It depends on the form of the concentration profile in the membrane phase. The *β* model parameter shows the form of the concentration profile of the electrolyte in the membrane. If *β* is equal to 1, the form of the concentration profile in the membrane phase is linear, and the P^*^ value does not depend on the electrolyte concentration. If *β* is higher than 1, the form of the concentration profile in the membrane phase is convex, and P^*^ increases with the electrolyte solution concentration. If *β* is lower than 1, the concentration profile in the membrane phase is concave, and P^*^ decreases with the electrolyte solution concentration.

Thus, the experimental data on the concentration dependences of the conductivity and diffusion permeability of a membrane allows for defining the volume fraction and conductivity of the conducting phases, their relative position, and the membrane selectivity.

## 4. Results and Discussion

### 4.1. Study of the Membrane Surface by SEM

**Figure 6:**
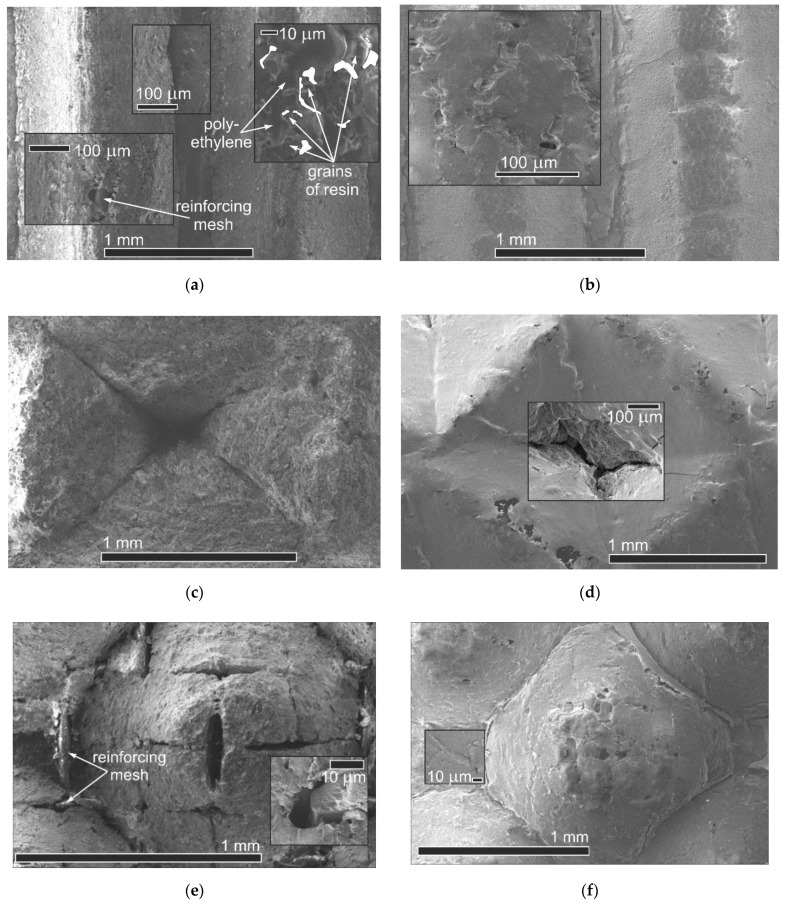
The SEM images of the profiled membrane surface for a single layer (**a**,**c**,**e**) and bilayer (**b**,**d**,**f**) membranes: a, b: No. 1; c, d: No. 2; e, f: No. 3.

An important aspect is to identify the effect of profiling and subsequent covering of an MF-4SK layer on the change in the microstructure of the membrane surface. Figure 6 shows the membrane surfaces with different profile shapes before and after the application of an MF 4SK layer. It can be seen that for membranes with profiles 1 and 3, the reinforcing mesh has come into view on the membrane surface. Also, there are rough defects both in the base of the profile elements and their surfaces. In the case of profile No. 2, the elements of the reinforcing mesh are not visible on the membrane surface. However, the major defects in the shape of a depressed tetrahedral pyramid with dimensions up to 120 μm × 200 μm (Figure 6c) appear in the depth of formed elements of the profile. Such changes can lead to a significant increase in diffusion transfer. The casting of the MF-4SK layer on the membrane surfaces with profiles No. 1 and No. 3 leads to the covering of most of the defects with a polymer film (Figure 6b,f). However, in the case of profile No. 2, it is not always possible to ensure complete coverage of all elements of the profile with the MF-4SK film, and rather large defects remain at the top of the depressed tetrahedral pyramid. The decrease in the depth of the profile element, in this case, would probably ensure complete coverage of the membrane surface with the MF-4SK film.

Analysis of the membrane surface images obtained with 550× magnification shows that individual grains of ion-exchange resin surrounded by amorphous polyethylene are clearly visible on the membrane surface without MF-4SK film. After the MF-4SK film casting, the membrane surface becomes more uniform, and it is no longer possible to visualize individual grains of the ion-exchange material. These results are in good agreement with the results of studying the surface of a heterogeneous MK-40 membrane covered with an MF-4SK layer by SEM and AFM methods [37,69], showing that the appearance of the MF-4SK film on the surface of a heterogeneous membrane causes its homogenization. Analysis of the membranes’ cross-section images permits the establishment of the thickness of the modifier film, which is 14–25 µm.

It should be noted that these studies were performed for dry membrane samples. The formation of cavities and channels transporting the ions and solvent takes place in IEMs during their swelling as a result of solvent sorption. That is why the structure of wet and dry membranes is quite different. In addition, the results relate mainly to the membrane surface and do not lead to the conclusion of possible changes inside the membrane. On the whole, the deposition of the MF 4SK film on the profiled membrane surface leads to an almost complete disappearance of large defects on the surface for all samples and a significant decrease in their microheterogeneity. Such changes should provide an improvement in the membrane transport properties, including a decrease in nonselective diffusion transfer of electrolytes through large pores. 

### 4.2. Diffusion Permeability of the Ion-Exchange Membranes

The diffusion permeability is the main property of IEM because it depends on the membrane selectivity and affects the efficiency of membrane processes. As for ED, the lower the diffusion permeability of IEM is, the higher the ED efficiency is because the migration and diffusion fluxes run in opposite directions. The reason for the appearance of the diffusion flow during ED is the concentration polarization phenomena. In the case of RED, the diffusion flow across the IEM is a consequence of a concentration gradient between the surrounding chambers. It leads to a decrease in the concentration gradient, which has a negative effect. However, the high diffusion flow across the IEM is positive for the diffusion dialysis process. The study of the diffusion permeability for modified and initial membranes in the NaCl solutions is performed.

**Figure 7:**
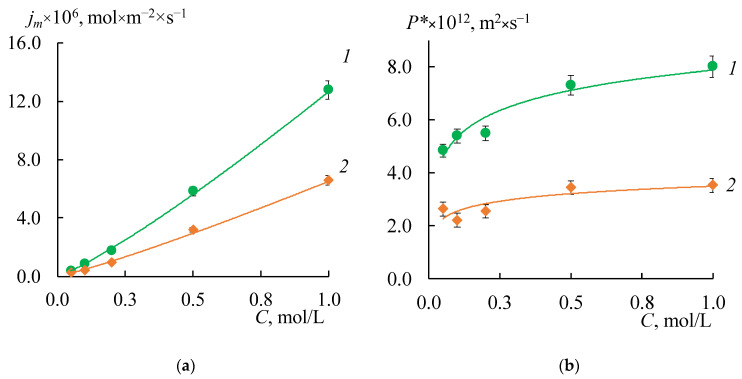
The concentration dependencies of the diffusion flux densities (**a**) and the differential diffusion permeability coefficient (**b**) of the MK-40 (1) and MA-41 (2) membranes in the NaCl solutions.

Figure 7 shows the concentration dependences of the differential coefficients of the diffusion permeability and diffusion fluxes for both initial membranes. These curves have a typical shape for IEM. The values of P^*^ for the MK-40 membranes are about two times higher than for the MA-41 membranes.

**Figure 8:**
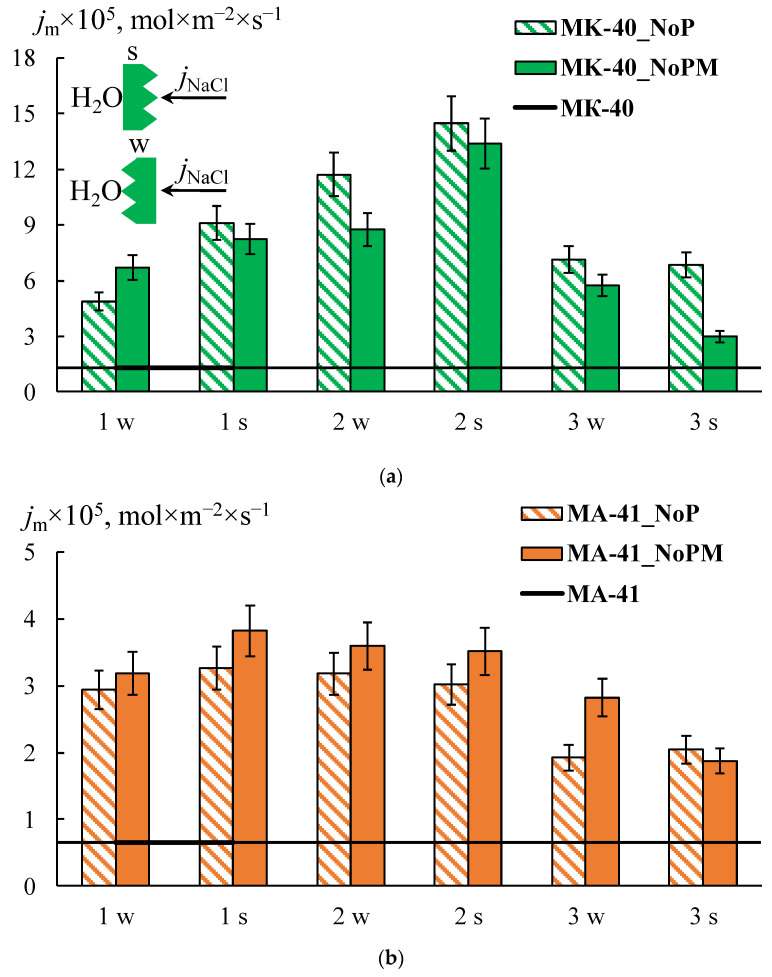
The flux density for the initial MK-40 (**a**) and MA-41 (**b**) membranes (line), the profiled membrane (shaded rectangles), and the profiled membranes with an MF-4SK layer (one-colored rectangles) in a 1 M NaCl solution. The number on the abscissa axis corresponds to the number of the sample profile in Table 1, and the letter corresponds to the orientation of the sample.

As expected, the formation of a profile on the membrane surface in all cases leads to an increase in the diffusion flux through the membrane due to the appearance of structural defects between the ion-exchange resin and polyethylene on the profiled membrane surface. Besides, the asymmetry of the P^*^ and *j_m_* values depending on the orientation of the membrane surface to the electrolyte flux (Figure 8a,b, shaded rectangles) is found. These effects are more pronounced for the MK-40 membrane. The highest value is obtained for the MK-40_2P sample in the case of s orientation: the *j_m_* values are 12–18 times higher than that for the initial membrane. The *j_m_* values for the MK-40_1P sample in the case of the w orientation and for the MK-40_3P sample in both orientations are in the range from 0.2 × 10^−5^ to 7 × 10^−5^ mol/(m^2^ × s) in the entire concentration range of the studied solutions, that is 4–15 times higher than the original membrane. The lowest diffusion permeability for MK-40_1P membranes in s-orientation is slightly higher than for the MK-40_3P membrane in both orientations. Thus, the MK-40_3P membrane has the lowest diffusion permeability. As for MA-41 membranes, the *j*_m_ values for MA-41_1P and MA-41_2P samples in both orientations are in the range from 0.2 × 10^−5^ to 3.3 × 10^−5^ mol/(m^2^ × s) in the entire concentration range of the studied solutions, that is 4–8 times higher compared to the original membrane. The MA-41_3P sample has lower diffusion permeability values than MA-41_1P and MA-41_2P membranes. However, the *j_m_* values remain about three times higher compared to the original MA-41 membrane.

The MF-4SK layer on the surface of the profiled MK-40 membrane leads to a decrease in the diffusion permeability: P^*^ and *j_m_* values for all samples are about 1.2–2.6 times lower than for a single-layer profiled MK-40 membrane. This is a predictable result, which is associated with a decrease in membrane surface defects formed during their profiling [27]. It is not obvious why the MF-4SK layer on the MK-40_1P membrane surface leads to an increase in the diffusion permeability for the w-orientation. This result can be explained by the fact that the measurement error of diffusion through profiled membranes is higher compared to smooth ones and by the general surface heterogeneity of a heterogeneous membrane, that in some cases leads to a significant difference in experimental characteristics (about 10%) for different samples of the same membrane.

**Figure 9:**
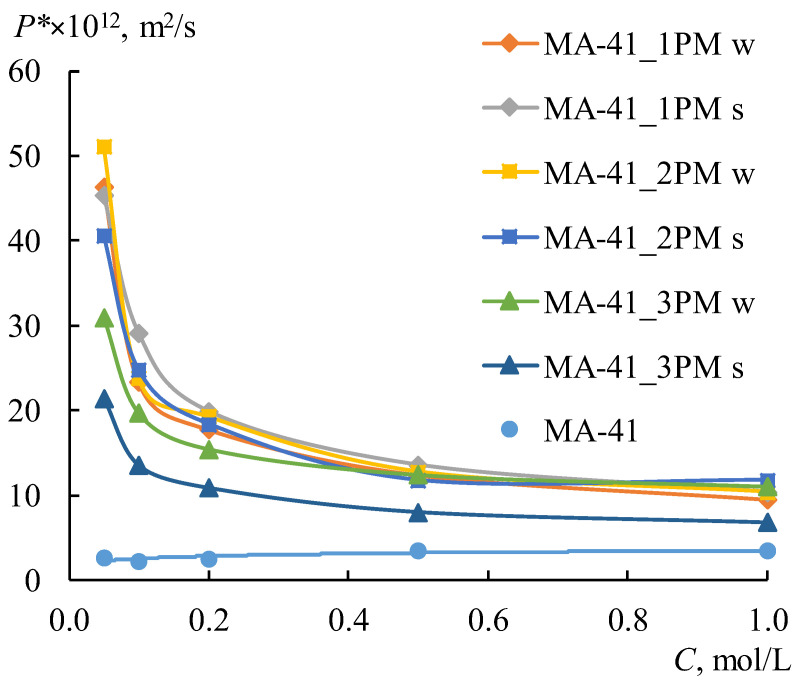
Concentration dependencies of the diffusion permeability of the initial MA-41 membrane and profiled bilayer membranes.

Unlike the MK-40 membrane, the MF-4SK layer on the MA-41 membrane surface leads to an increase in the P^*^ and *j_m_* values for all samples, with the exception of the MA-41_3PM membrane for the s-orientation. The differences between these values for MA-41_3PM and MA-41_3P membranes do not exceed the experimental error (Figure 8b). These results are more pronounced for diluted solutions: their *j_m_* values in 0.1 M NaCl are about 3–5 times higher than those of profiled membranes without the MF-4SK layer. Besides, the shape of the concentration dependencies of the diffusion permeability coefficients changes after casting the MF-4SK layer on the membranes profiled surfaces for all samples: these dependencies became decreasing (Figure 9). As usual, such shapes of P^*^-*C* dependence have been observed for solutions containing multiple charged ions, which are the counter-ions for the membrane [70]. The possible reasons for such a significant change in the shape of the concentration dependence of the diffusion permeability of membranes are discussed in Section 4.4. in detail.

The increases in diffusion permeability can be explained by the formation of a mosaic membrane structure. In this case, the flux of cations passes through the cation-exchange parts of the membrane, and the flux of anions passes through anion-exchange parts of the membrane [71,72]. The bilayer AEMs having a mosaic structure are prospective for the separation of electrolytes, which contain monovalent/divalent ions [73].

The asymmetry of the diffusion permeability for all of the profiled membranes was found to be more pronounced in diluted solutions. However, it was not possible to reveal a clear dependence. At the same time, for the MK-40 membranes, in most cases, the value of *P*_s_ is higher than of *P*_w,_ and the ratio of these values ranges from 2.8 to 1.0. The exceptions are MK-40_3PM and MA-41_3PM samples. The dependence is reversed for MK-40_3PM membrane in solutions with concentrations above 0.5 M, and the asymmetry coefficient is 0.6–0.5. The asymmetry coefficient is 0.7–0.8 in the entire range of studied concentrations for the MA-41_3PM membrane. 

The analysis of the experimental results shows that the minimum diffusion permeability values are observed for the MK-40_3PM and MA-41_3PM samples, which makes these membranes the most promising for use in the ED and RED. At the same time, these samples have a higher diffusion permeability in comparison with the original membranes, which allows us to conclude that their use in the processes of dialysis separation of solutions is promising.

### 4.3. Conductivity of the Ion-Exchange Membranes

**Figure 10:**
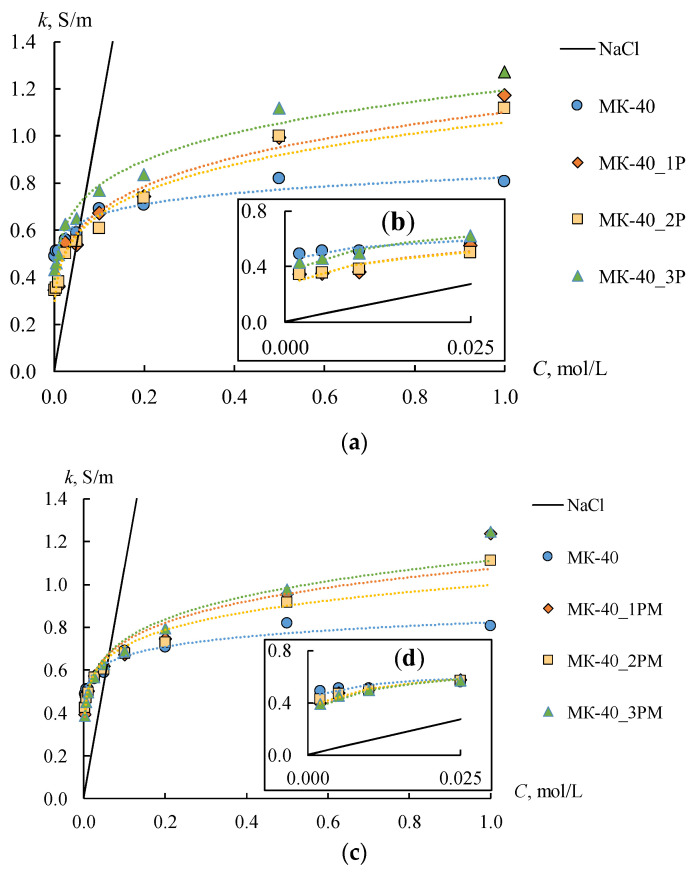
Concentration dependences of conductivity (**a**,**c**) and their initial sections (**b**,**d**) for smooth, profiled (**a**,**b**) and bilayer profiled (**c**,**d**) MK-40 membranes in NaCl solutions.

The conductivity is the most important property of the IEM, which influences the efficiency of the electromembrane processes. Figure 10 shows the concentration dependencies of the conductivity of initial, profiled, and bilayer profiled membranes. The *k_m_* of the MK-40 membrane is two times higher than that of MA-41, which is caused by the lower ion-exchange capacity of the MA-41 membrane. The conductivity of all profiled CEMs increases in the range of solution concentrations above 0.2 M compared to the smooth one. The highest conductivity values are observed for the MK-40_3P sample. The conductivity of this membrane is 1.6 times higher in 1 M NaCl solution than of the initial membrane. The conductivity of MK-40_2P and MK-40_1P samples increases by about 1.4 times compared to the initial MK-40 membrane in the same solution. This fact indirectly indicates an increase in the volume fraction of the solution phase in the membrane. The gel phase has a higher conductivity in the diluted solutions compared to the conductivity of a solution phase due to the high concentration of fixed ions and, consequently, the counter-ions. Consequently, an increase in the volume fraction of the solution phase in an IEM should lead to a decrease in its conductivity in the diluted solutions. This effect is observed for profiled membranes in the low concentration region (up to 0.025 M NaCl). The conductivity of the initial membrane remains significantly higher than that of profiled membranes (Figure 10b). The solution conductivity inside the membrane becomes higher than during the membrane gel phase in concentrated solutions. Therefore, the overall conductivity will be higher for samples with a larger volume fraction of the solution phase in the membrane. It is observed for profiled CEMs.

**Figure 11:**
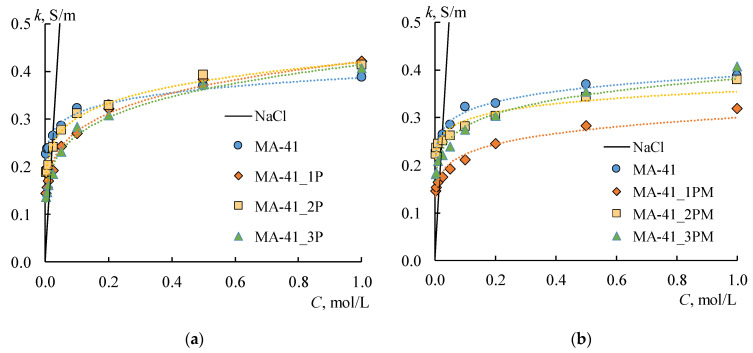
Concentration dependences of conductivity for initial, profiled (**a**), and bilayer profiled (**b**) MA-41 membranes in NaCl solutions.

Similar changes in conductivity are observed for the profiled MA-41 AEMs (Figure 11a), but they are less pronounced. Thus, an increase in the conductivity of profiled AEMs is observed in a sodium chloride concentration higher than 0.5 M and does not exceed 9%. The shape of the profile also does not have any significant effect on the value of conductivity. The conductivity values of the MA-41_2P membrane turn out to be the highest in comparison with the other two profiled membranes in solution concentrations below 0.5 M. The conductivity of the MA-41_2P sample is 19% lower than the initial one in 0.002 M NaCl. As for the MA-41_1P and MA 41_3P samples, their conductivity decreases by 36–40% compared to the initial membrane. On the whole, the conductivity values for MA-41_1P and MA-41_3P samples are approximately the same.

The main expected result from the application of the MF-4SK layer to the profiled surface of the membrane is the coverage of large defects, which have been formed as a result of membrane profiling. In general, the results of the membrane surface visualization, as well as the study of the diffusion permeability of all membranes, confirmed the achievement of this result. It is also well known that the conductivity of the MF-4SK membrane is much higher than that of the MK-40 and MA-41 membranes. So, the *k_m_* of the MF-4SK, MK-40, and MA-41 membranes are about 1 S/m, [74] 0.69 S/m, and 0.32 S/m in 0.1 M NaCl, respectively. Thus, an increase in conductivity can be expected after the deposition of the MF-4SK layer on the membrane surface. The conductivity of the smooth anion-exchange heterogeneous membrane with an MF-4SK layer is about 10–15% higher than that of the initial one [64]. However, the increase in the conductivity values after casting the MF 4SK layer on the profiled surfaces is observed only for MK-40_1PM, MK-40_2PM, MA 41_2PM, and MA-41_3PM membranes in diluted solutions.

The more pronounced increases in conductivity are found for CEMs. The *k_m_* of the MK 40_1PM and MK-40_2PM membranes are higher, by about 30–35%, than the MK-40_1P and MK-40_2P membranes. As for AEMs, the increase in the *k_m_* value is less than 25% and 30% for the MA-41_2PM and MA-41_3PM membranes, respectively. The conductivity of the MK-40_3PM membrane is the same as that of the MK-40_3P one. These facts are explained by the small thickness of the MF-4SK layer. The conductivity of all the profiled CEMs, which have been modified by the MF-4SK layer, remains higher than that of the initial membrane in concentrated solutions.

Another effect is observed for the MA-41_1PM membrane. The conductivity of this membrane decreases when the MF-4SK layer appears on the profiled surface. The higher the concentration of the solution, the more essential the conductivity decrease is (Figure 11b, orange rhombi). This unexpected effect can be explained as follows. A large number of defects have appeared on the surface of this membrane due to profiling. As a result, the area of contact between the MF-4SK layer and the grains of the ion-exchange resin greatly increases. In this case, some of the fixed MF-4SK groups can compensate for the charge of the amino groups of the MA-41 membrane, which leads to the observed decrease in conductivity.

### 4.4. Selectivity and Transport-Structural Parameters of the Ion-Exchange Membranes

**Figure 12:**
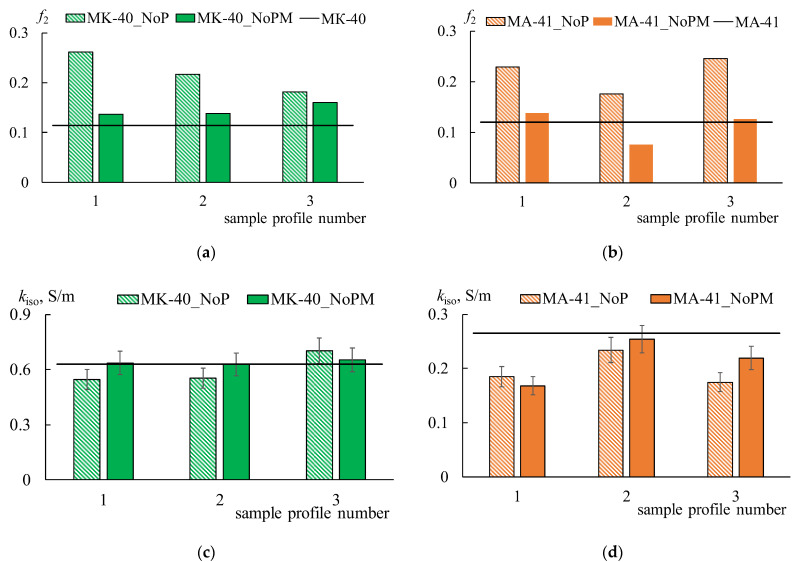
The volume fraction of a gel phase (*f*_1_ parameter) (**a**,**b**) and the conductivity of the gel phase (**c**,**d**) for initial (line), profiled (shaded rectangles), and bilayer profiled membranes (one-colored rectangles). The number on the abscissa axis corresponds to the number of the sample profile in Table 1.

The transport-structural parameters of the IEMs are obtained based on the concentration dependences of the diffusion permeability and membrane conductivity by the procedure described in Section 3. The *α*, *β*, *f*_1_, *f_2,_* and *k_iso_* parameters for all membranes are estimated (Table 2, Figure 12). The *G* parameter is calculated by the Formula (10) for all CEMs. As for AEMs, the G parameter is determined only for initial and profiled membranes due to the abnormal shape of P^*^-*C* dependencies for bilayer profiled membranes. In this case, the values of the *β* parameter are less than one.

Creating a profile on the MK-40 membrane’s surface leads to the formation of structural defects between the ion-exchange resin particles and polyethylene, independent of what method is used. This effect is accompanied by a decrease in volume fractions of the gel phase (*f*_1_ parameter), an increase in volume fractions of the solution phase (*f*_2_ parameter), and a change in the relative position of these phases. The increase in the *α* parameter indicates the growth in parallel connection of conducting phases.

The application of the MF-4SK layer on profiled membrane leads to the filling of the structure defects both on the surface and in the membrane volume. It is confirmed by a change in the values of the *f*_1_, *f_2,_* and *α* parameters: if the changes in the structure affected only the surface, the volume fractions of the conducting phases and their mutual arrangement would not change. However, the values of the *f*_1_ and *α* parameters increase. Moreover, the values of the *α* parameter for modified membranes are almost independent of the profile shape and orientation of the membrane to the electrolyte solution flux.

The form of the electrolyte concentration profile inside the membrane phase changes due to the change in the membrane structure as a result of pressing. The membrane orientation towards the electrolyte flux also affects the concentration profile form, which changes from convex in the initial MK-40 membrane to concave in the MK-40_3PM membrane. It is indicated by the corresponding changes in the value of the *β* parameter: 1.17 for the MK-40 membrane and 0.78 for the MK-40_3PM membrane in the case of its orientation with the modified side to the electrolyte flux. At the same time, surface modification leads to an increase in the *β* parameter for the MK-40_1PM and MK-40_2PM samples, regardless of orientation.

The *G* parameter is the most sensitive to the membrane profile form, modification, and orientation of the membrane to the salt flow. Differences in the values of this parameter for the MK-40_2P single and MK-40_2PM bilayer profiled samples exceed two orders of magnitude. However, it is not possible to identify the regularities between the change in this parameter and the mentioned factors.

In the case of pressing the MA-41 membrane, the character of changes in the *f*_1_ and α parameters are similar to the MK-40 membrane. At the same time, the modification of the surface of the AEM with a cation-exchange polymer leads to a decrease in the *β* value by almost two times as a result of the formation of a mosaic structure. It corresponds to a concave form of the concentration profile and a decrease in the differential coefficient of diffusion permeability with an increase in the concentration of electrolyte solutions. It should be noted that the *β* and *G* parameters strongly depend on both the pressing method and orientation of the membrane towards the salt flow.

**Figure 13:**
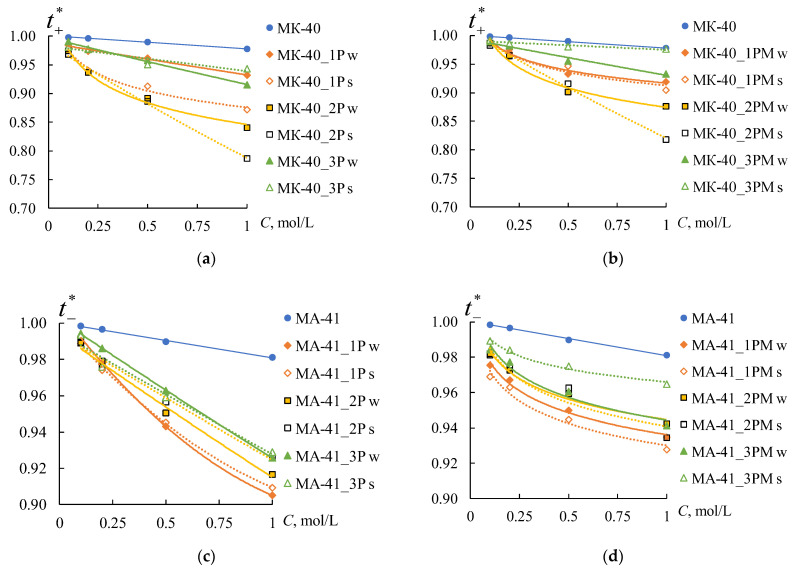
The concentration dependencies of the counter-ion transport number in the MK-40 CEMs (**a**,**b**) and MA-41 AEMs (**c**,**d**), where (**a**,**c**) are profiled membranes, (**b**,**d**) are bilayer profiled membranes.

In general, the observed increase in the membrane diffusion permeability coefficients after profiling and coating by MF-4SK indicates a decrease in membrane selectivity. Figure 13 shows the concentration dependences of the counter-ion transport numbers in the membranes, which are calculated using the procedure described in Section 2.2. 

The values of the transport numbers of counter-ions in the membrane decrease with an increase in the electrolyte solution concentration for all samples. It is consistent with the known data [48,74,75]. The reduction of $t_{i}^{*}$ in both initial membranes is no more than 2%. Creating a profile on the membrane’s surface leads to a decrease in the values of $t_{i}^{*}$ for all samples. The values of the counter-ion transport numbers in the profiled membranes depend on the orientation of profiled surface towards the counter-ion flux. This effect is more pronounced for the MK-40 membranes than for the MA-41 ones.

The profile forms affect the decrease in $t_{i}^{*}$ only for the MK-40 membranes. The most significant decrease in counter-ion transport numbers is observed for the sample with profile No. 2, less significant for the sample with profile 1, and the smallest for the sample with profile No. 3. 

As expected, applying the MF-4SK layer on the profiled membrane surface leads to an increase in the values of $t_{i}^{*}$ for all membrane samples (Figure 13b,d). The maximum values of $t_{i}^{*}$ are observed for the samples with profile No. 3 for both types of membranes.

Thus, the study of the conductivity, diffusion permeability, and selectivity of the single and bilayer profiled membranes has shown that the most promising samples for the application in the RED are the MK-40_3PM and MA-41_3PM membranes with profile No. 3. 

### 4.5. Current-Voltage Curves of the Ion-Exchange Membranes

**Figure 14:**
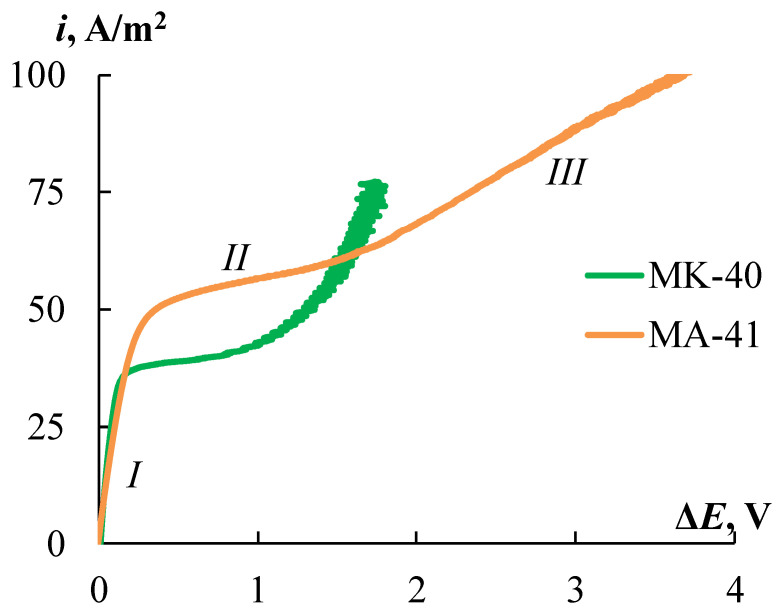
The CVCs of the initial MK-40 and MA-41 membranes in a 0.05 M NaCl solution: *I*—the ohmic section; *II*—the plateau of limiting current; *III*—the overlimiting region.

Figure 14 shows the CVCs for the initial MK-40 and MA-41 membranes. The CVCs have a typical form that has three regions: the ohmic section, the plateau of the limiting current, and the overlimiting region. The current and potential drop are linearly increasing at the ohmic section (Figure 14, section *I*). Since the transport numbers of counter-ions in the membrane are higher than in solution, the concentration of counter-ions in the depleted diffusion layer decreases. An increase in current leads to a decrease in the concentration of counter-ions in the diffusion layer, and at a certain value of the current density, called the limiting current density, their concentration tends to zero. In this case, a further increase in the current density due to an increase in the flux of electrolyte ions with an increase in the applied voltage becomes impossible, and a plateau of the limiting current appears on the CVC (Figure 14, section *II*). An increase in the current density above the limiting value is associated with the development of coupled phenomena of concentration polarization, the main of which are electroconvection and water splitting [35,76,77,78,79,80,81]. These phenomena correspond to the overlimiting section of the CVC (Figure 14, section *III*).

The *i_lim_* value for the MK-40 membrane is 36% lower than for MA-41 (Figure 14). The electromembrane systems conductivities, which are defined as the slope of the ohmic section of the CVC (d*i*/d*E_ohmic_*), are 1.8 times higher for the MK-40 membrane compared to the MA-41 one. This is consistent with the results obtained from the AC conductivity measurements given in Section 4.3. The plateau length is significantly longer for the MA-41 membrane, which may be associated with the later development of electroconvection due to the higher catalytic activity of secondary and tertiary amino groups in the water splitting [82]. It is known that water splitting prevents the development of electroconvection [81,83]. A less significant contribution of electroconvection to the overlimiting transfer is also indicated by the lower conductivity of the electromembrane system with the MA-41 membrane in the overlimiting region. 

**Figure 15:**
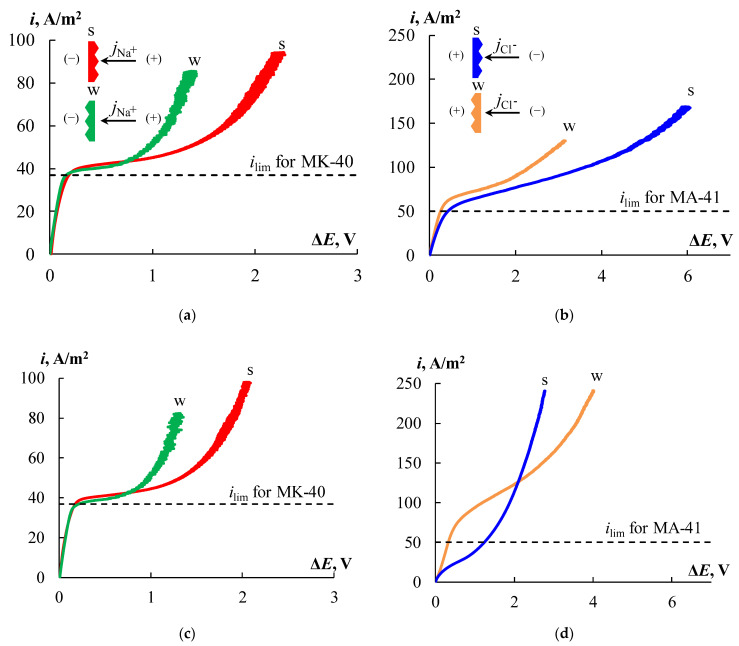
The CVCs of the profiled (**a**,**b**) and profiled with MF-4SK layers (**c**,**d**) MK-40 (**a**,**c**) and MA-41 membranes (**b**,**d**) in a 0.05 M NaCl solution with different membrane orientations to the flow of counter-ions indicated by the mark “s” and “w”. The dashed line shows the limiting current density for the initial membranes.

Formation of the profile on the surface of the CEMs and AEMs leads to changes in CVCs. Figure 15 shows the CVCs of the profiled and bilayer profiled membranes with profile No. 3. The main difference between the CVCs of the profiled CEMs from the initial smooth ones is the asymmetry of the plateau length. When the profiled surface faces the counter-ion flux, the limiting current plateau length is about 20% longer than that for the initial membrane. The plateau length is 30% less than that of the initial membrane in the case of opposite orientation. The values of *i_lim_* are equal for all CEMs. The deposition of an MF-4SK layer on the profiled surface of the MK-40 membrane practically does not lead to a change in the CVC compared with the single-layer profiled membrane.

Profiling the surface of the AEM, as well as of the CEM, leads to an asymmetry in the plateau length. The effect of asymmetry in the case of the profiled AEM is more pronounced, and for both orientations of the membrane, an increase in the plateau length is observed in comparison with the initial MA-41 membrane. The plateau length increases by 2.5 times when the profiled surface is oriented toward the counter-ion flux and only by 20% for the opposite orientation. In addition, the limiting current density of profiled MA-41_3P membrane is 10% higher for orientation with the profiled side toward the counter-ion flux and is 20% higher for opposite orientation than of the initial one.

The invariability of the value of the limiting current density for profiled CEMs and the increase in this value for the AEM in comparison with the initial membrane are interesting. There are a number of possible reasons that could lead to such changes which should be considered. The limiting current density is calculated by the formula

(20) $$i_{lim}=\frac{DCF}{(t_{i}^{*}-t_{i})\delta }+\frac{P^{*}FC}{l(t_{i}^{*}-t_{i})},$$

where *δ* is the thickness of the diffusion layer [84]. It is obvious that the decreases in the transport number of the counter-ion in the membrane, the membrane thickness, the thickness of the diffusion layer, and the increase in the diffusion permeability after profiling lead to the rise of the *i_lim_* value. A change in the membrane surface area should also lead to a change in the *i_lim_* value. The growth of the limiting current is observed only for AEMs. The transport numbers of the counter-ion in all profiled membranes are slightly decreased in the region of diluted solutions (Figure 13). The growth of the membrane diffusion permeability after profiling is more pronounced for CEMs. So, these facts cannot fully explain the observed effects. Neither the change in the membrane area nor the thickness of the diffusion layer after profiling can be determined. But it can be assumed that the observed effects are determined by the relationship between the change in the surface area of the membrane, its thickness in the regions corresponding to the protrusions and depressions of the relief, and the thickness of the depleting diffusion layer. However, this assumption requires additional research.

It has been proposed that creating a profile on the membrane surface would provide favorable conditions for earlier development of electroconvection. However, obtained results show a later transition of the electromembrane system from the limiting state to the overlimiting one in all cases when the membrane profiled surface is turned to the counter-ion flux. It can be assumed that the large dimensions of profile elements complicate the formation of electroconvective vortices at the membrane surface.

Figure 15d shows the CVCs for the MA-41_3PM membrane, which are very different from the other CVCs, especially in the case of orientation by a profiled surface with an MF-4SK layer towards counter-ion flux (s-orientation). The *i_lim_* value for s-orientation is two and three times lower than the initial MA-41 membrane and opposite to the w-orientation of the MA-41_3PM membrane, respectively. This effect could be explained by the formation of bipolar junctions between positively charged amino groups of the AEM and negatively charged sulfo-groups of the MF-4SK layer that leads to catalytic water splitting. The same behavior for an asymmetric bipolar membrane consisting of layers of unequal thickness is described elsewhere [65,85,86]. Such membranes can be effective in the separation of single and multiply charged ions [43,87] or organic and inorganic ions [88]. The positive effect is an increase in the conductivity of the electromembrane system for the MA-41_3PM membrane in the overlimiting state compared to initial membranes.

The CVCs for the CEMs showed that the single and bilayer profiled membranes have the same transport properties until the current density is limited and slightly deteriorates in the overlimiting current mode. The profiled AEM has higher values of the limiting current density in comparison with a smooth membrane which makes it promising for use in ED processes.

### 4.6. Application of Profiled and Bilayer Profiled Membranes in Reverse Electrodialysis

**Figure 16:**
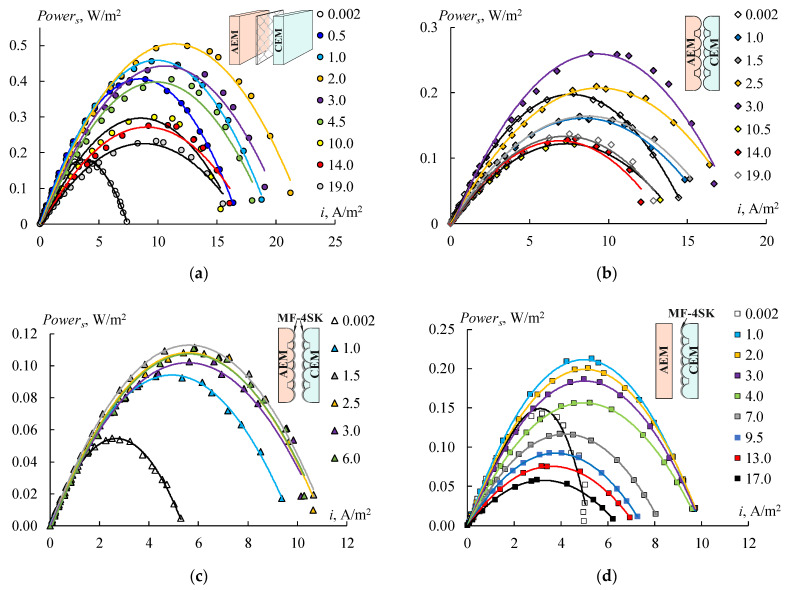
Power density versus current density at different concentrations (g/L) of NaCl in diluted solution for different kinds of membranes into the RED stack: initial smooth MA-41 and MK-40 membranes (**a**), profiled MA-41_3P and MK-40_3P membranes (**b**), profiled bilayer MA-41_3PM and MK-40_3PM membranes (**c**), initial smooth MA-41 and profiled modified MK-40_3PM membranes (**d**). The concentration values are shown in each figure.

**Figure 17:**
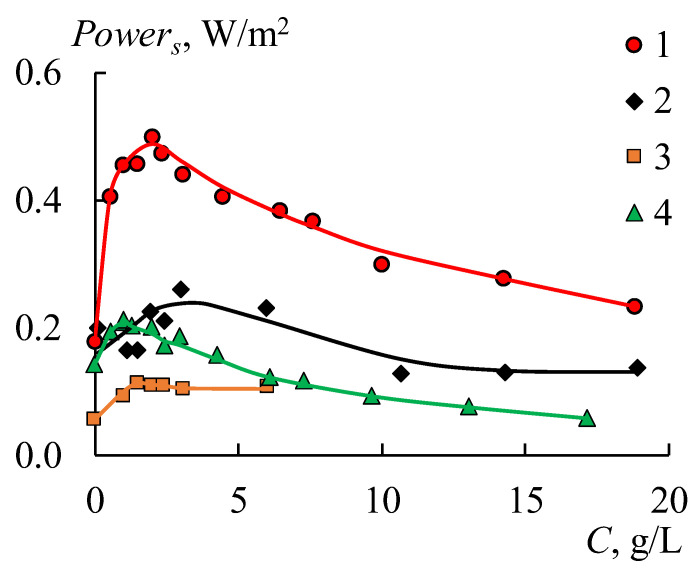
Influence of diluted solution concentration on power density for different membrane stacks with the initial smooth MK-40 and MA-41 membranes (1), the profiled MK-40_3P and MA-41_3P membranes (2), the profiled bilayer MK-40_3PM and MA-41_3PM membranes (3), the profiled modified MK-40_3PM and the initial smooth MA-41 membranes (4).

Figure 16 shows the power density dependencies of RED on the current density for different concentrations of the diluted solution. Four types of the RED stack with different membranes have been tested. The first stack consists of initial smooth MK 40 and MA 41 membranes (Figure 16a), the second one consists of profiled MK 40_3P and MA 41_3P membranes (Figure 16b), the third one consists of bilayer profiled MK 40_3PM and MA 41_3PM membranes (Figure 16c), and the fourth one consists of bilayer profiled MK 40_3PM membranes and initial smooth MA 41 membranes (Figure 16d). All of these dependencies have a typical form and allow us to determine the maximum power density value for each studied concentration of a diluted solution (Figure 17).

An increase in the concentrations of sodium chloride in a diluted solution leads to an increase in power density in all cases at the region of low concentrations due to a growth in the solution conductivity (Figure 17). The power density begins to decrease when the concentration of the diluted solution reaches a certain value due to the reduction in the concentration gradient between the chambers. The values of these concentrations are different for each RED stack in the range of 1–3 g/L. Creating a profile on the membrane surface leads to a two-fold decrease in the obtained power density as a result of declining their selectivity (Figure 16b and Figure 17, black rhombi). Using bilayer profiled membranes leads to a decrease in the maximum power density to 0.11 W/m^2^ (Figure 16c and Figure 17, orange rectangles) because of the appearance of the bipolar contact between the MF-4SK layer and the substrate of the MA-41 membrane. To eliminate this negative effect, the initial smooth MA-41 membrane is used as an AEM. The obtained power grows up to 0.21 W/m^2^ (Figure 16c) when using a modified profiled MK 40_3PM membrane and the initial MA 41 one. But this value is lower than the power density for initial smooth membranes (Figure 16a). The maximum value of the power for the MK 40_3PM and the initial MA 41 membrane has been obtained at a diluted solution with a salinity of 1 g/L (Figure 17, green triangle). While in the case of both profiled membranes, the maximum value has been obtained at the concentration of 3 g/L (Figure 17, black rhombi) due to the lower electrical resistance of the RED stack with profiled membranes.

It should be noted that the discussed power density values are gross values, and they do not take into account the pumping power, which is several times lower in the case of channels formed by profiled membranes than by inert spacers [6,24,28].

Despite the obvious negative effect of using obtained profiled membranes in the process of RED, it can be seen that the properties of IEMs have a significant effect on the efficiency of RED. At the same time, previously obtained results showed a great efficiency of using these membranes in the ED process in comparison with smooth membranes [37]. A comparison of the obtained results with those known from the literature for profiled membranes [6,24,26] suggests that the main disadvantage of the membranes obtained in this work is the exclusively large size of the profile elements compared to the thickness of the membrane itself. Optimization of the shape and size of the profile elements, as well as the profiling procedure, shall be done in such a way that it allows, on the one hand, the low hydrodynamic resistance of the channel without the use of inert spacers and, on the other hand, maintaining the transport characteristics of the material at the level of initial smooth membranes. In the future, it will allow obtaining profiled membranes that improve the characteristics of RED.

## 5. Conclusions

In the present study, single and bilayer profiled membranes with three different types of surface profiles based on the commercial heterogeneous ion-exchange MK-40 and MA-41 membranes have been obtained. The bilayer membrane consists of the substrate profiled membrane and an MF-4SK film, which is a Nafion-type homogeneous CEM, on the profiled surface. It is shown that membrane profiling leads to an increase in their diffusion permeability due to the appearance of large defects on their surface. The study of the microstructure of the surface of profiled membranes before and after deposition of the MF-4SK film by SEM has shown that large defects appear on the membrane surface during profiling and then are filled partially or completely with the MF-4SK film. The application of the MF-4SK film on the profiled surface of CEMs reduces their diffusion permeability by 1.5–2 times. However, in the case of applying the homogeneous film to profiled AEMs, an increase in the diffusion permeability coefficients is observed. A study of the concentration dependences of the membranes’ conductivity shows that profiling leads to an increase in their conductivity in the region of concentrated solutions.

The transport-structural parameters and the transport numbers of counter-ions are calculated for all studied membranes based on the concentration dependences of the conductivity and diffusion permeability using a microheterogeneous model. The decrease of volume fractions of a gel phase, increase of volume fractions of a solution phase, and a change in the relative position of these phases are found for the profiled IEM. The counter-ion transport numbers are lower for the profiled membranes than for the smooth ones. Applying an MF-4SK layer on the profiled membranes surfaces leads to an increase in the counter-ion transport numbers, but these values are about 2–10% lower than those of the initial membrane.

Profiling the surface of the IEMs leads to asymmetry in the length of the plateau of the limiting current: the highest values are observed when the profiled surfaces are turned to the counter-ions flux. But creating the profile on the AEM surface has a greater effect on the parameters of the CVCs in contrast with the CEM. So, the limiting current density practically does not change for the single and bilayer profiled CEM compared with the initial smooth MK-40 one. But the increase of the limiting current density for the profiled AEM is found for both membrane orientations, which makes them promising for use in ED processes. The shape of the CVC of the bilayer AEM is typical for the asymmetric bipolar membrane due to the formation of bipolar junctions between positively charged amino groups of the AEM and negatively charged sulfo-groups of the MF-4SK layer, which makes them promising for use in the separation of single and multiply charged ions.

The study of RED with the initial and different types of profiled membranes in a wide range of diluted solutions concentrations of sodium chloride is carried out. The use of the profiled membrane in RED leads to a decrease in the power density in all studied solution concentrations. This effect is more pronounced for the bilayer profiled membranes: the maximum power density value, in this case, was five times lower in comparison with the initial membranes. Thus, it can be noted that obtained membranes can be effectively used in the processes of dialysis and ED; however, RED requires optimization of the size of the profile elements.

## Figures and Tables

**Table 1:** The physical-chemical properties of initial and profiled single layer and bilayer membranes.

Membrane	Water Uptake, %	*l*, µm	$n_{w},\;\frac{\mathrm{mol}\;H_{2}O}{\mathrm{mol}\;\mathrm{fixed}\mathrm{ions}}$	IEC, mmol/g_dry_
MK-40	36	534	15.0	2.12 ± 0.09
MK-40_1P	43	565	19.7	
MK-40_2P	41	537	18.2	
MK-40_3P	42	538	19.2	
MK-40_1PM	41	542	18.4	
MK-40_2PM	41	540	18.1	
MK-40_3PM	41	550	18.3	
MA-41	26	441	23.4	0.84 ± 0.08
MA-41_1P	33	462	32.8	
MA-41_2P	30	470	27.6	
MA-41_3P	31	495	29.3	
MA-41_1PM	32	489	30.9	
MA-41_2PM	30	460	28.2	
MA-41_3PM	30	490	28.2	

**Table 2:** The transport-structural parameters of initial, single, and bilayer profiled membranes.

Membrane	Orientation	α	*β*	G×10^−17^, m^5^/(mol × s)
CEMs				
MK-40		0.36	1.17	18.35
MK-40_P1	w	0.31	1.09	5.94
	s	0.41	1.02	2.75
MK-40_P2	w	0.52	1.00	0.18
	s	0.53	1.02	27.83
MK-40_P3	w	0.43	1.12	84.41
	s	0.53	0.81	–
MK-40_P1M	w	0.50	1.15	576.81
	s	0.55	1.09	380.44
MK-40_P2M	w	0.53	1.19	1556.41
	s	0.54	1.23	1042.31
MK-40_P3M	w	0.47	1.09	57.87
	s	0.49	0.78	–
AEMs				
MA-41		0.31	1.22	7.96
MA-41_P1	w	0.27	1.25	58.19
	s	0.31	1.08	2.76
MA-41_P2	w	0.39	1.01	0.05
	s	0.39	0.96	–
MA-41_P3	w	0.23	1.26	22.44
	s	0.26	1.14	4.28
MA-41_P1M	w	0.48	0.61	–
	s	0.50	0.56	–
MA-41_P2M	w	0.61	0.63	–
	s	0.67	0.55	–
MA-41_P3M	w	0.48	0.75	–
	s	0.45	0.70	–

## Data Availability

The data presented in this study are available on request from the corresponding author.

## References

[1] Jang J., Kang Y., Han J.-H., Jang K., Kim C.-M., Kim I.S. (2020). Developments and future prospects of reverse electrodialysis for salinity gradient power generation: Influence of ion exchange membranes and electrodes. Desalination.

[2] Jwa E., Yoon K., Mok Y.S., Oh J.H., Han J., Jeung Y.C., Hwang K.S., Kim H., Jeong N., Nam J.Y. (2022). Enhanced electrochemical disinfection of domestic aquaculture wastewater with energy production in reverse electrodialysis. Aquaculture.

[3] Jianbo L., Chen Z., Kai L., Li Y., Xiangqiang K. (2021). Experimental study on salinity gradient energy recovery from desalination seawater based on RED. Energy Convers. Manag..

[4] Ramasamy G., Rajkumar P.K., Narayanan M. (2021). Generation of energy from salinity gradients using capacitive reverse electro dialysis: A review. Environ. Sci. Pollut. Res..

[5] Filimonova A.A., Chichirov A.A., Chichirova N.D. (2021). The utilization of highly mineralized liquid waste from a chemical desalination water treatment plant of a TPP with the generation of electrical energy by reverse electrodialysis. Membr. Membr. Technol..

[6] Vermaas D.A., Saakes M., Nijmeijer K. (2011). Power generation using profiled membranes in reverse electrodialysis. J. Memb. Sci..

[7] Tian H., Wang Y., Pei Y., Crittenden J.C. (2020). Unique applications and improvements of reverse electrodialysis: A review and outlook. Appl. Energy.

[8] Daniilidis A., Vermaas D.A., Herber R., Nijmeijer K. (2014). Experimentally obtainable energy from mixing river water, seawater or brines with reverse electrodialysis. Renew. Energy.

[9] Pattle R.E. (1954). Production of electric power by mixing fresh and salt water in the hydroelectric pile. Nature.

[10] Jin D., Xi R., Xu S., Wang P., Wu X. (2021). Numerical simulation of salinity gradient power generation using reverse electrodialysis. Desalination.

[11] Wang W., Zhang Y., Tan M., Xue C., Zhou W., Bao H., Hon Lau C., Yang X., Ma J., Shao L. (2022). Recent advances in monovalent ion selective membranes towards environmental remediation and energy harvesting. Sep. Purif. Technol..

[12] Zhang W., Yan H., Wang Q., Zhao C. (2022). An extended Teorell-Meyer-Sievers theory for membrane potential under non-isothermal conditions. J. Memb. Sci..

[13] Altıok E., Kaya T.Z., Othman N.H., Kınalı O., Kitada S., Güler E., Kabay N. (2022). Investigations on the effects of operational parameters in reverse electrodialysis system for salinity gradient power generation using central composite design (CCD). Desalination.

[14] Kang S., Li J., Wang Z., Zhang C., Kong X. (2022). Salinity gradient energy capture for power production by reverse electrodialysis experiment in thermal desalination plants. J. Power Sources.

[15] Zhang Y., Wu X., Xu S., Leng Q., Wang S. (2022). A serial system of multi-stage reverse electrodialysis stacks for hydrogen production. Energy Convers. Manag..

[16] Wu X., Zhang X., Xu S., Gong Y., Yang S., Jin D. (2021). Performance of a reverse electrodialysis cell working with potassium acetate−methanol−water solution. Energy.

[17] Chanda S., Tsai P.A., Choi J.-Y., Yang S.-C. (2021). Renewable power generation by reverse electrodialysis using an ion exchange membrane. Membranes.

[18] Hulme A.M., Davey C.J., Tyrrel S., Pidou M., McAdam E.J. (2021). Transitioning from electrodialysis to reverse electrodialysis stack design for energy generation from high concentration salinity gradients. Energy Convers. Manag..

[19] Novitsky E.G., Grushevenko E.A., Vasilevsky V.P., Volkov A. (2020). V Studying the possibilities of generating electric-power by reverse electrodialysis of monoethanolamine aqueous solutions. Membr. Membr. Technol..

[20] Guo Z., Ji Z., Zhang Y., Yang F. (2018). Effect of ions (K^+^, Mg^2+^, Ca^2+^ and SO_4_^2−^) and temperature on energy generation performance of reverse electrodialysis stack. Electrochim. Acta.

[21] Cui W.Z., Ji Z.Y., Tumba K., Zhang Z.D., Wang J., Zhang Z.X., Liu J., Zhao Y.Y., Yuan J.S. (2022). Response of salinity gradient power generation to inflow mode and temperature difference by reverse electrodialysis. J. Environ. Manage..

[22] Pawlowski S., Huertas R.M., Galinha C.F., Crespo J.G., Velizarov S. (2020). On operation of reverse electrodialysis (RED) and membrane capacitive deionisation (MCDI) with natural saline streams: A critical review. Desalination.

[23] Pawlowski S., Geraldes V., Crespo J.G., Velizarov S. (2016). Computational fluid dynamics (CFD) assisted analysis of profiled membranes performance in reverse electrodialysis. J. Memb. Sci..

[24] Pawlowski S., Rijnaarts T., Saakes M., Nijmeijer K., Crespo J.G., Velizarov S. (2017). Improved fluid mixing and power density in reverse electrodialysis stacks with chevron-profiled membranes. J. Memb. Sci..

[25] Güler E., Elizen R., Saakes M., Nijmeijer K. (2014). Micro-structured membranes for electricity generation by reverse electrodialysis. J. Memb. Sci..

[26] Długołecki P., Dabrowska J., Nijmeijer K., Wessling M. (2010). Ion conductive spacers for increased power generation in reverse electrodialysis. J. Memb. Sci..

[27] Zabolotskii V.I., Loza S.A., Sharafan M. (2005). V Physicochemical properties of profiled heterogeneous ion-exchange membranes. Russ. J. Electrochem..

[28] Gurreri L., Battaglia G., Tamburini A., Cipollina A., Micale G., Ciofalo M. (2017). Multi-physical modelling of reverse electrodialysis. Desalination.

[29] Kotoka F., Merino-Garcia I., Velizarov S. (2020). Surface modifications of anion exchange membranes for an improved reverse electrodialysis process performance: A review. Membranes.

[30] Nagarale R.K., Gohil G.S., Shahi V.K. (2006). Recent developments on ion-exchange membranes and electro-membrane processes. Adv. Colloid Interface Sci..

[31] Pourcelly G., Nikonenko V.V., Pismenskaya N.D., Yaroslavtsev A.B. (2012). Applications of Charged Membranes in Separation, Fuel Cells, and Emerging Processes. Ionic Interactions in Natural and Synthetic Macromolecules.

[32] Stenina I., Golubenko D., Nikonenko V., Yaroslavtsev A. (2020). Selectivity of transport processes in ion-exchange membranes: Relationship with the structure and methods for its improvement. Int. J. Mol. Sci..

[33] Singh R., Kim D. (2022). Ultrafast ion-transport at hierarchically porous covalent-organic membrane interface for efficient power production. Nano Energy.

[34] Golubenko D.V., Van der Bruggen B., Yaroslavtsev A.B. (2021). Ion exchange membranes based on radiation-induced grafted functionalized polystyrene for high-performance reverse electrodialysis. J. Power Sources.

[35] Nikonenko V.V., Pismenskaya N.D., Belova E.I., Sistat P., Huguet P., Pourcelly G., Larchet C. (2010). Intensive current transfer in membrane systems: Modelling, mechanisms and application in electrodialysis. Adv. Colloid Interface Sci..

[36] Zabolotsky V.I., Novak L., Kovalenko A.V., Nikonenko V.V., Urtenov M.H., Lebedev K.A., But A.Y. (2017). Electroconvection in systems with heterogeneous ion-exchange membranes. Pet. Chem..

[37] Loza S.A., Zabolotsky V.I., Loza N.V., Fomenko M.A. (2016). Structure, morphology, and transport characteristics of profiled bilayer membranes. Pet. Chem..

[38] Choi J.H., Kim S.H., Moon S.H. (2001). Heterogeneity of ion-exchange membranes: The effects of membrane heterogeneity on transport properties. J. Colloid Interface Sci..

[39] Akberova E.M., Vasil’eva V.I. (2020). Effect of the resin content in cation-exchange membranes on development of electroconvection. Electrochem. Commun..

[40] Akberova E.M., Vasil’eva V.I., Zabolotsky V.I., Novak L. (2018). Effect of the sulfocation-exchanger dispersity on the surface morphology, microrelief of heterogeneous membranes and development of electroconvection in intense current modes. J. Memb. Sci..

[41] Sheldeshov N.V., Zabolotskii V.I., Loza S.A. (2014). Electric conductivity of profiled ion exchange membranes. Pet. Chem..

[42] Vasil’eva V., Goleva E., Pismenskaya N., Kozmai A., Nikonenko V. (2019). Effect of surface profiling of a cation-exchange membrane on the phenylalanine and NaCl separation performances in diffusion dialysis. Sep. Purif. Technol..

[43] Sata T. (2000). Studies on anion exchange membranes having permselectivity for specific anions in electrodialysis—Effect of hydrophilicity of anion exchange membranes on permselectivity of anions. J. Memb. Sci..

[44] Zhao Z., Cao H., Shi S., Li Y., Yao L. (2016). Characterization of anion exchange membrane modified by electrodeposition of polyelectrolyte containing different functional groups. Desalination.

[45] Weinstein J.N., Leitz F.B. (1976). Electric power from differences in salinity: The dialytic battery. Science.

[46] Apel P.Y., Bobreshova O.V., Volkov A.V., Volkov V.V., Nikonenko V.V., Stenina I.A., Filippov A.N., Yampolskii Y.P., Yaroslavtsev A.B. (2019). Prospects of membrane science development. Membr. Membr. Technol..

[47] Yaroslavtsev A.B., Stenina I.A. (2021). Current progress in membranes for fuel cells and reverse electrodialysis. Mendeleev Commun..

[48] Berezina N.P., Kononenko N.A., Dyomina O.A., Gnusin N.P. (2008). Characterization of ion-exchange membrane materials: Properties vs structure. Adv. Colloid Interface Sci..

[49] Demina O.A., Shkirskaya S.A., Kononenko N.A., Nazyrova E.V. (2016). Assessing the selectivity of composite ion-exchange membranes within the framework of the extended three-wire model of conduction. Russ. J. Electrochem..

[50] Luo T., Abdu S., Wessling M. (2018). Selectivity of ion exchange membranes: A review. J. Memb. Sci..

[51] Berezina N., Gnusin N., Dyomina O., Timofeyev S. (1994). Water electrotransport in membrane systems. Experiment and model description. J. Memb. Sci..

[52] Yeager H.L., O’Dell B., Twardowski Z. (1982). Transport properties of Nafion® membranes in concentrated solution environments. J. Electrochem. Soc..

[53] Arnold R., Swift D.A. (1967). Electro-osmosis and hydrogen-ion transport in cation-exchange membranes. Aust. J. Chem..

[54] Nikonenko V.V., Pis’menskaya N.D., Volodina E.I. (2005). Rate of generation of ions H+ and OH− at the ion-exchange membrane/dilute solution interface as a function of the current density. Russ. J. Electrochem..

[55] Greben’ V.P., Rodzik I.G. (2005). Selectivity of transport of sodium, magnesium, and calcium ions through a sulfo-cationite membrane in mixtures of solutions of their chlorides. Russ. J. Electrochem..

[56] Le X.T. (2012). Concentration polarization and conductance of cation exchange membranes in sulfuric acid and alkaline sulfate media. J. Memb. Sci..

[57] Krol J.J., Wessling M., Strathmann H. (1999). Chronopotentiometry and overlimiting ion transport through monopolar ion exchange membranes. J. Memb. Sci..

[58] Titorova V.D., Mareev S.A., Gorobchenko A.D., Gil V.V., Nikonenko V.V., Sabbatovskii K.G., Pismenskaya N.D. (2021). Effect of current-induced coion transfer on the shape of chronopotentiograms of cation-exchange membranes. J. Memb. Sci..

[59] Tian C., Kristiansen K.R., Kjelstrup S., Barragán V.M. (2022). Two methods for determination of transport numbers in ion-exchange membranes. Int. J. Thermophys..

[60] Gnusin N.P., Berezina N.P., Kononenko N.A., Dyomina O.A. (2004). Transport structural parameters to characterize ion exchange membranes. J. Memb. Sci..

[61] Zabolotsky V.I., Nikonenko V.V. (1993). Effect of structural membrane inhomogeneity on transport properties. J. Memb. Sci..

[62] Berezina N.P., Kononenko N.A., Demina O.A., Gnusin N.P. (2004). Model approach for describing the properties of ion-exchange membranes. Polym. Sci. Ser. A.

[63] (1972). Ion-Exehahge Membranes. Method for Determination of Total and Equilibrium Exchange Capacity.

[64] (1972). Ion-Exchange Membranes. Method for Determination of Moisture.

[65] Melnikov S., Shkirskaya S. (2019). Transport properties of bilayer and multilayer surface-modified ion-exchange membranes. J. Memb. Sci..

[66] Demina O.A., Berezina N.P., Sata T., Demin A.V. (2002). Transport-structural parameters of domestic and foreign anion-exchange membranes. Russ. J. Electrochem..

[67] Karpenko L.V., Demina O.A., Dvorkina G.A., Parshikov S.B., Larchet C., Auclair B., Berezina N.P. (2001). Comparative study of methods used for the determination of electroconductivity of ion-exchange membranes. Russ. J. Electrochem..

[68] Andreeva M., Loza N., Kutenko N., Kononenko N. (2020). Polymerization of aniline in perfluorinated membranes under conditions of electrodiffusion of monomer and oxidizer. J. Solid State Electrochem..

[69] Andreeva M.A., Gil V.V., Pismenskaya N.D., Nikonenko V.V., Dammak L., Larchet C., Grande D., Kononenko N.A. (2017). Effect of homogenization and hydrophobization of a cation-exchange membrane surface on its scaling in the presence of calcium and magnesium chlorides during electrodialysis. J. Memb. Sci..

[70] Demina O.A., Kononenko N.A., Falina I.V., Demin A.V. (2017). Theoretical estimation of differential coefficients of ion-exchange membrane diffusion permeability. Colloid J..

[71] Fukuda T., Yang W., Yamauchi A. (2003). KCl transport mechanism across charged mosaic membrane in KCl–sucrose mixed system. J. Memb. Sci..

[72] Summe M.J., Sahoo S.J., Whitmer J.K., Phillip W.A. (2018). Salt permeation mechanisms in charge-patterned mosaic membranes. Mol. Syst. Des. Eng..

[73] Linder C., Kedem O. (2001). Asymmetric ion exchange mosaic membranes with unique selectivity. J. Memb. Sci..

[74] Falina I., Loza N., Loza S., Titskaya E., Romanyuk N. (2021). Permselectivity of cation exchange membranes modified by polyaniline. Membranes.

[75] Auclair B., Nikonenko V., Larchet C., Métayer M., Dammak L. (2002). Correlation between transport parameters of ion-exchange membranes. J. Memb. Sci..

[76] Belloň T., Polezhaev P., Vobecká L., Svoboda M., Slouka Z. (2019). Experimental observation of phenomena developing on ion-exchange systems during current-voltage curve measurement. J. Memb. Sci..

[77] Belloň T., Slouka Z. (2020). Overlimiting behavior of surface-modified heterogeneous anion-exchange membranes. J. Memb. Sci..

[78] Choi J.H., Lee H.J., Moon S.H. (2001). Effects of electrolytes on the transport phenomena in a cation-exchange membrane. J. Colloid Interface Sci..

[79] Rubinstein I., Zaltzman B. (2015). Equilibrium electroconvective instability. Phys. Rev. Lett..

[80] Rubinstein I., Zaltzman B. (2000). Electro-osmotically induced convection at a permselective membrane. Phys. Rev. E—Stat. Phys. Plasmas Fluids Relat. Interdiscip. Top..

[81] Zabolotsky V.I., Nikonenko V.V., Pismenskaya N.D., Laktionov E.V., Urtenov M.K., Strathmann H., Wessling M., Koops G.H. (1998). Coupled transport phenomena in overlimiting current electrodialysis. Sep. Purif. Technol..

[82] Zabolotskii V.I., Shel’deshov N.V., Gnusin N.P. (1988). Dissociation of water molecules in systems with ion-exchange membranes. Russ. Chem. Rev..

[83] Nikonenko V.V., Mareev S.A., Pis’menskaya N.D., Uzdenova A.M., Kovalenko A.V., Urtenov M.K., Pourcelly G. (2017). Effect of electroconvection and its use in intensifying the mass transfer in electrodialysis (Review). Russ. J. Electrochem..

[84] Kononenko N.A., Demina O.A., Loza N.V., Dolgopolov S.V., Timofeev S.V. (2021). Theoretical and experimental investigation of limiting diffusion current in the systems with modified perfluorinated membranes containing sulfonic acid groups. Russ. J. Electrochem..

[85] Wilhelm F.G., Pünt I., Van der Vegt N.F.A., Strathmann H., Wessling M. (2002). Asymmetric bipolar membranes in acid-base electrodialysis. Ind. Eng. Chem. Res..

[86] Pärnamäe R., Mareev S., Nikonenko V., Melnikov S., Sheldeshov N., Zabolotskii V., Hamelers H.V.M., Tedesco M. (2021). Bipolar membranes: A review on principles, latest developments, and applications. J. Memb. Sci..

[87] Vaselbehagh M., Karkhanechi H., Takagi R., Matsuyama H. (2015). Surface modification of an anion exchange membrane to improve the selectivity for monovalent anions in electrodialysis—Experimental verification of theoretical predictions. J. Memb. Sci..

[88] Melnikov S.S., Nosova E.N., Melnikova E.D., Zabolotsky V.I. (2021). Reactive separation of inorganic and organic ions in electrodialysis with bilayer membranes. Sep. Purif. Technol..

